# Supplementary material for: Analytical Validation of an Immunohistochemical 7-Biomarker Prognostic Assay (immunoprint®) for Early-Stage Cutaneous Melanoma in Archival Tissue of Patients with AJCC v8 T2–T3 Disease
Source: Diagnostics (Basel). 2023 Sep 29;13(19):3096. doi: 10.3390/diagnostics13193096 (PMC10572486; doi:10.3390/diagnostics13193096)
Supplement: Supplementary file 1 [file diagnostics-13-03096-s001.zip › diagnostics-2639332-supplementary.pdf]

Supplementary Information

Supplementary Tables

Supplementary Table S1. Antibodies used for S-100 staining and 7-marker signature assay biomarker staining

Antibody	Supplier, Location
Mouse anti-S100, monoclonal	Zytomed Systems, Berlin, Germany
Mouse anti-CD20, monoclonal	Zytomed Systems, Berlin, Germany
Rabbit anti-human BCL-X (EP94), monoclonal	BioSB, Santa Barbara, CA, USA
Rabbit anti-COX-2 (SP21), monoclonal	Cell Marque, Sigma-Aldrich, Rocklin, CA, USA
Rabbit anti-human Bax, polyclonal	Cell Signaling Technology, Danvers, MA, USA
Rabbit anti-human β-Catenin, polyclonal	Cell Signaling Technology, Danvers, MA, USA
Rabbit anti-PTEN, monoclonal	Cell Signaling Technology, Danvers, MA, USA
Rabbit anti-MTAP, polyclonal	ChromoTek and Proteintech Germany, Planegg-Martinsried, Germany

Supplementary Table S2. Overview of patient screening and inclusion targets

**Group or T-stage subgroup**	**# Expected To Be Screened***			**Inclusion targets (# of patients)**		
	**High-Risk**	**Low-Risk**	**Total**	**High-Risk**	**Low-Risk**	**Total**
**T2**	9	7	16	5	6	11
**T3**	7	5	12	5	4	9
**Total**	16	12	28	10	10	20

***** The inclusion target was specimens from 20 discrete patients, 10 of each to be 7-marker signature high-risk and low-risk, respectively. The expected number to be screened was based on an projected 60% of patients with T2/T3 tumors having 7-marker signature high-risk status and 40%, low-risk status, as seen in clinical validation studies [12,13,15]. Additionally, the number to be screened was based on the projected rate of samples failing S-100 staining confirmation of melanoma or failing quality control examination at the central laboratory.
